# Antiseptic Dental Surgery

**Published:** 1895-12

**Authors:** A. W. Harlan

**Affiliations:** Chicago


### Antiseptic Dental Surgery.

BY A. W. HARLAN, M.D., D.D.S., CHICAGO.

Abstract of a paper read by title at the American Dental Association, 1895.

It is not the normal pulp we are called upon to treat surgically, except in rare instances, but the uncovered pulp, or the pulp which is receding, or the one exposed at the apex of a root, or the pulp which is gradually being transformed into spurious dentine, or one with a fungus growth upon it. * * * *

What constitutes true antiseptic surgery of the pulp? First, the understanding of antiseptic surgery. It is no longer surgery which only excludes the causes of putrefaction; we may now include, rather, in the term all those methods of wound treatment in which the growth and fermentative action of the lower forms of organisms (bacteria) are more or less impeded. Instruments used are to be sterilized by heat or by use of agents which will not fail to destroy all particles from the air or moisture that they are brought in contact with.

My own observations lead me to the conclusion that the pulp to be preserved must be kept from contact with saliva or mixed fluids of the mouth. If water is used upon the surface of a pulp it must be sterilized before such use. Filtration is not sterilization. The water must be boiled, and all instruments to be thoroughly sterilized must be cleaned and afterward boiled in a sodium carbonate solution, two to three per cent., or silico-fluoride, a saturated solution, 1–144.

The pulp being in a closed cavity, after exposure by disease or intentionally, is to be removed surgically with all necessary precautions, to insure freedom from infection of the pericementum at the apex of a root; the general principle being that no septic matter should be allowed access to a pulp canal after the surgical or mechanical removal of a pulp.

All operations on roots of teeth under the gum and between the tooth root and the alveolar process will be more uniformly successful when they are made with surgically clean instruments.

No syringe point should be introduced into the pericementum or the opening that is not surgically clean. Dirt, dried blood, mucus, paste, or any septic matter adhering to a needle will not be rendered aseptic by washing in a chemical disinfectant in any strength solution, unless it destroys such substances as fire will, or superheated steam in an oven.

Such dressings as are used in the mouth should be made of gauze impregnated with boracic acid, iodoform or iodin solution. Such dressings should be as dry as possible. * * * Moisture, which is hard to exclude from the mouth and around the jaws, is a *bete noir* for the dentist. It is on this account that the teachings of aseptic surgery cannot be fully utilized in oral operations.

It is the duty of every surgeon to practice antiseptic surgery to the fullest possible extent and to make and keep records of such work to encourage others to do likewise for the benefit of our fellow-men.

